# Discovering Pair-Wise Genetic Interactions: An Information Theory-Based Approach

**DOI:** 10.1371/journal.pone.0092310

**Published:** 2014-03-26

**Authors:** Tomasz M. Ignac, Alexander Skupin, Nikita A. Sakhanenko, David J. Galas

**Affiliations:** 1 Luxembourg Centre for Systems Biomedicine, Esch-sur-Alzette, Luxembourg; 2 Pacific Northwest Diabetes Research Institute, Seattle, Washington, United States of America; 3 National Center for Microscopy and Imaging Research, University of California San Diego, La Jolla, California, United States of America; Tel Aviv University, Israel

## Abstract

Phenotypic variation, including that which underlies health and disease in humans, results in part from multiple interactions among both genetic variation and environmental factors. While diseases or phenotypes caused by single gene variants can be identified by established association methods and family-based approaches, complex phenotypic traits resulting from multi-gene interactions remain very difficult to characterize. Here we describe a new method based on information theory, and demonstrate how it improves on previous approaches to identifying genetic interactions, including both synthetic and modifier kinds of interactions. We apply our measure, called interaction distance, to previously analyzed data sets of yeast sporulation efficiency, lipid related mouse data and several human disease models to characterize the method. We show how the interaction distance can reveal novel gene interaction candidates in experimental and simulated data sets, and outperforms other measures in several circumstances. The method also allows us to optimize case/control sample composition for clinical studies.

## Introduction

The rapid progress of sequencing technology, in both accuracy and cost, has enabled comprehensive Genome-Wide Association Studies (GWAS) which have identified many genetic contributions to complex phenotypes in humans (see www.genome.gov) and continues to be productive. The primary focus of GWAS is the reliable extraction of relevant genetic markers such as SNPs and indels that are associated with a complex phenotype. Numerous studies of gene regulatory networks, protein interaction networks, and other biological networks have made it clear, however, that genetic interactions are widespread and therefore important for full genetic analysis [1]. Moreover, complex, non-additive genetic interactions are very common and are potentially critical in determining phenotypes [2]–[5]. It is clear that the missing heredity problem has at least part of its solution in the interaction effects [6]. GWAS and similar studies, including QTL analyses, use statistical methods based on correlation or likelihood and are aimed primarily at detecting single locus effects on a phenotype. These statistical methods usually assume additive models of multi-gene effects, representing a compound effect of multiple genes on a phenotype as a sum of the effect of each individual gene [7], [8].

Recently, new methods, based on information theory, that are aimed specifically at detecting complex, non-additive interactions have been proposed [9]–[11]. Typically these methods consist of two major components, a measure of non-additive interaction defined *via* information theory, such as interaction information [9] or “total correlation information” [10], and an algorithm, such as multifactor dimensionality reduction [12] that searches for interactions across a large set of genetic markers. In this paper we focus primarily on the first component, a normalized interaction measure, which we call Interaction Distance (ID), leaving the specific, detailed strategy of application of the measures outside of the scope of this paper.

Information theory based methods have the advantage of being intrinsically model-free and parsimonious and thus offer an unbiased and potentially statistically powerful approach to detection of genetic interactions. Moreover, even in situations where the sample size is not large enough for making statistically confident assessments, these methods can often be used to filter candidate interactions and to generate useful hypotheses [9]. The application of information theory based methods to human data, however, is still in its infancy and these methods are yet little tested. In this paper we show that small minor allele frequencies (MAFs) affect the current interaction measures sharply and, as a result, the downstream interaction search is strongly biased towards genetic markers with higher MAFs. Note that missing data and noise have similar biasing consequences.

Here, we propose a novel genetic interaction measure, called Interaction Distance, which uses information theory concepts with normalization and helps to address the problem of low MAFs. We show that our measure can improve the quality and robustness of the detection of modifier genes and synthetic effects on phenotype. We apply ID to several examples (yeast, mouse, and simulated human data) with increasing biological and computational complexity and evaluate the results using statistical permutation tests.

## Results

### The Approach

In human genetics a typical dataset consists of a large heterogeneous population characterized genetically by a series of polymorphisms or genetic markers, and phenotypically by a set of specific trait variations. To decipher the relationship between genetic markers and to construct a gene regulatory network we have to detect the dependency of the phenotypes on multiple variants in the population. Formally, detecting a genetic interaction corresponds to detecting a statistical dependence of 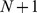 random variables, 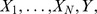 representing 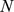 interacting genetic markers and the phenotypic trait. In the most common case of two interacting genes, the corresponding statistical dependence then involves three variables, 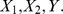 We distinguish three kinds of genetic interactions: *interactions between QTLs with marginal effects*, *modifier interactions*, and *synthetic interactions*. The first type spans only loci with individual effects on the phenotype and are the easiest to detect. Modifier interactions, on the other hand, link genetic markers, some of which exhibit marginal effects and some are markers with no effect by themselves, called modifiers of the significant loci (QTLs). The most difficult kind of interaction to detect is synthetic, which links genetic markers that have no marginal effect on the phenotype when present alone, but have an effect when present together. Formally, this corresponds to having no pairwise dependence between either gene variable, 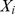, and the phenotype, 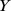, but a collective dependence among all three variables 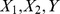. We use the term *genetic interaction* as a general short term referring to the interaction among genetic variants. Note that while we fully recognize the differences among the terms locus, QTL, modifier locus and gene, we use the term “genetic interaction” in the interest of brevity where the meaning is sufficiently clear.

Synthetic and modifier interactions are the focus of this work. In order to identify these interactions we have devised the Interaction Distance (ID) measure that extends the concept of interaction information (II) first proposed by McGill [13]. Interaction information has been applied in many fields and recently was successfully used in the analysis of genetic interactions [9], [10].

We define interaction information for three variables, 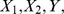 in terms of the mutual information 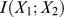 by the recursive relation
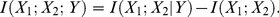
(1)


**Figure 1:**
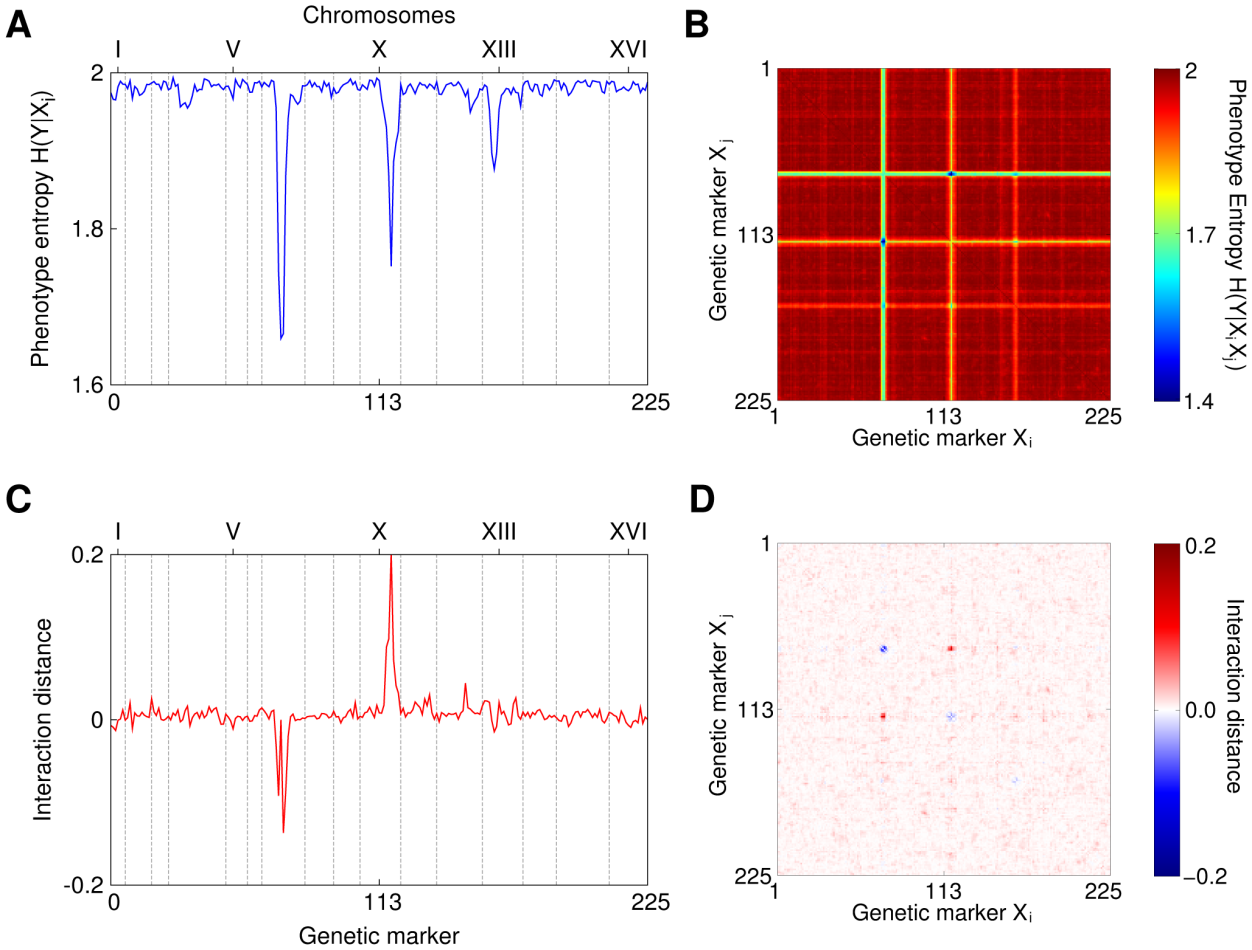
Genetic interactions in yeast. A) Conditional entropy of the phenotype given a single marker, 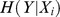. B) A heat map of conditional entropy 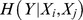 of the phenotype given two markers. Notice stripes caused by the markers with strongest single effects that make detection of pairs with small effect difficult, especially for a large number of markers. C) Interaction distance between marker 7.9, which is the marker with the strongest marginal effect, and every other marker. The “negative peak” shows that neighborhood markers contain redundant information. Most values fluctuates around zero since they do not interact with 7.9. D) A heat map of interaction distance for all pairs of markers and the phenotype, 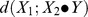. Note that there are no stripes anymore.

**Figure 2:**
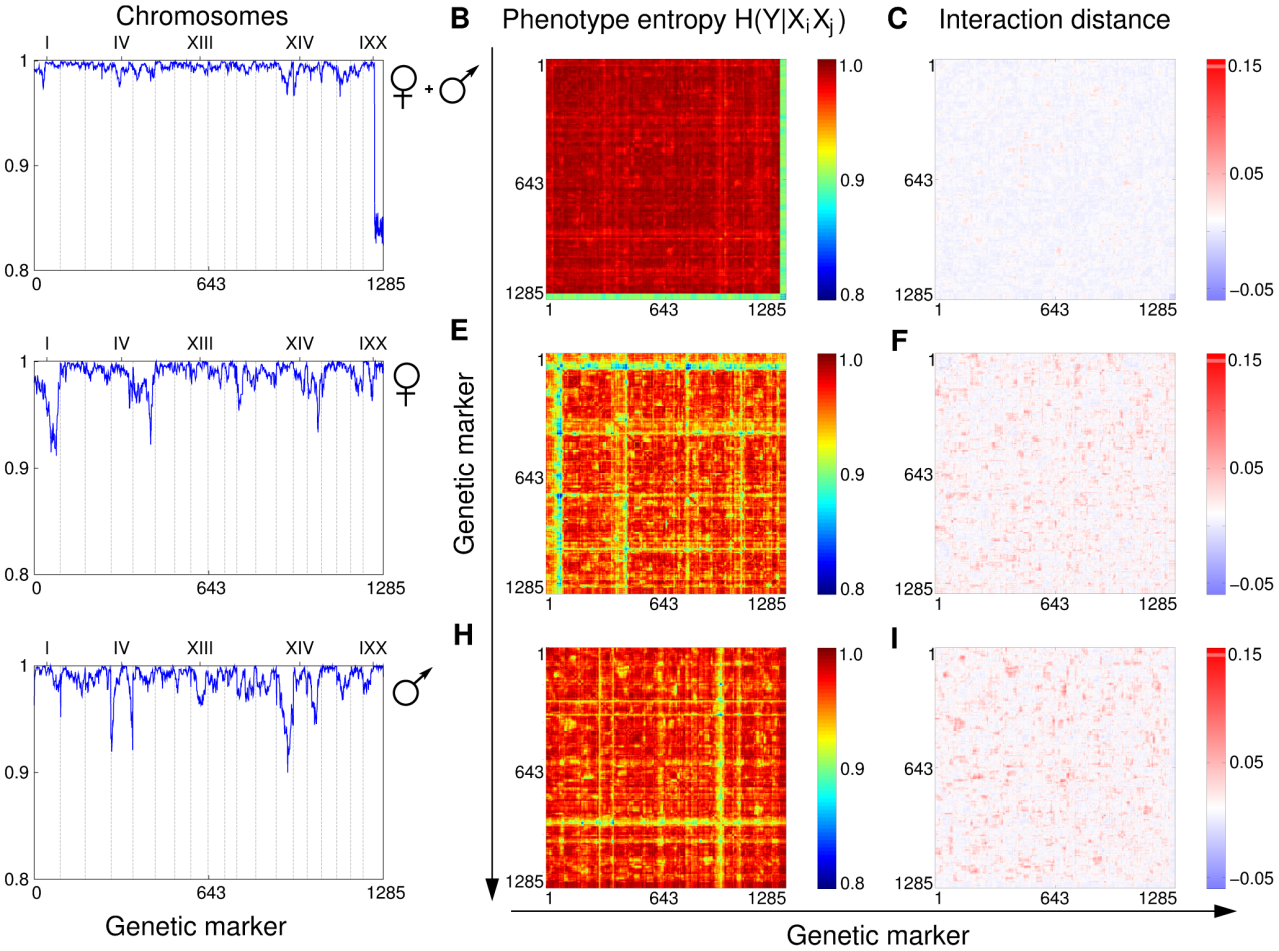
Genetic interactions in body weight phenotype of mouse. The first row, panels (A–C), shows results of the genetic analysis of the body weight for the full cohort of 303 mice (no sex division). Panel (A) shows conditional entropy 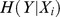, (B) shows 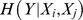 and (C) shows the interaction distance. The strongest effect on the phenotype in this case comes from markers located on the Y chromosome present only in males. This is expected since the weight is strongly correlated with the sex. Rows (D–F) and (G–I) show data for female and male subpopulations respectively. A comparison of (D) and (G) reveals sex specific QTLs affecting the phenotype. Panels (B), (E) and (H) exhibit the characteristic stripe pattern, which masks the more subtle synthetic and modifying interactions. Finally, (C), (F) and (I) plot the ID scores for all pairs of markers. The red spots in panels (C, F, I) and blue/yellow spots in panels (B, E, H) point out potentially interesting pairs, which are a subject of further investigation.

The conditional entropy, 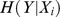, is a measure of dependence of 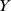 on a single variable 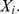 Similarly, 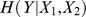 can measure dependence of 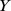 on a pair of variables 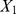 and 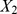. Although 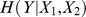 implicitly accounts for the interaction between 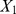 and 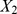, it is not suitable for interaction detection since its value corresponds to a cumulative effect of both individual variables as well as a pair of variables on the phenotype variable. This is illustrated by the application to yeast and mouse data in Figure 1 and Figure 2.

In contrast to the mutual information, which is a non-negative quantity, the interaction information can take both positive and negative values. With our sign convention positive values of the interaction information indicate presence of an informational “synergy”, or collective dependence among 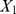, 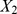, and 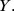 This means that the pair of variables 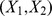 together contains more information about 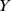 than 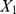 and 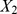 do when considered singly [13]–[15]. On the other hand, negative values suggest that 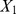 and 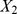 are redundant to some extent.

The interaction information can then, in principle, indicate synergy among variables. However, this measure is affected significantly when the entropy of the single variables is extreme. This situation arises in genetics, for example, when the minor allele frequency is particularly low, making the entropy also low. To account for this situation we adjust the interaction information by normalizing its components and define interaction distance as a new measure.

Recall that two random variables, 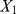 and 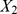, are said to have a synergistic effect on the third variable, 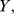 iff the amount of shared information between 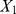 and 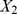 increases when conditioning on 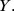 This is expressed in Equation 1. Note that the level of the shared information between variables in Equation 1 is expressed in terms of the unnormalized mutual information. We can replace the mutual information with the information distance [16] that normalizes the mutual information. The information distance between 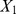 and 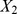 is defined as
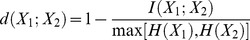
(2)and its conditional version is



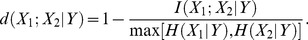
(3)Note that, when the correlation between pairs of variables grows, the mutual information grows, and the information distance decreases.

Following the notion of recursion as used in Equation 1 we use (2) and (3) to define the interaction distance (ID) between 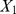 and 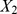 given 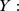


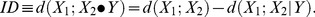
(4)


Unlike Equation 1, we subtract the conditional distance from the unconditional here to maintain the convention we adopted for the interaction information that a positive value of an interaction measure means a synergistic effect, while a negative value indicates redundancy. Note that, again unlike the interaction information, the interaction distance is not symmetric; i.e., it depends on the choice of the third variable. Three-way symmetry is an elegant property but it is not a necessary feature for such a measure. ID has been designed as a tool for genetic applications where the two genetic markers and the phenotype are clearly distinguished. The third variable in our case is the phenotype throughout the entire analysis. Then, ID is clearly symmetric with respect to 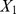 and 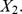


A striking difference between ID and the interaction information is that ID is a normalized measure. Normalization allows ID to adjust its information components so as to tackle the problem of small minor allele frequencies as we will illustrate.

The current section has presented the basic idea of interaction distance. More detailed discussion of the theoretical properties of ID and a further exposition of the theory are provided in the Methods section.

### Applications to Genetic Data

To illustrate our approach we applied ID to diverse genetic data sets. First we considered data from a relatively simple genetic system, the budding yeast *Saccharomyces cerevisae*, then we looked at a somewhat more complex system, the mouse. I.e., the genetics of the crosses are equivalent between yeast and mouse in general, but the number of states in the genetic loci of the progeny is two in yeast (haploid progeny are analyzed) and three in the mouse (diploid progeny). Finally we use models to simulate various characteristics of human genetics that are accessible using ID. The exposition and comparison of these applications is the central theme of this paper.

#### Analysis of data from a yeast cross

We analyzed a data set of the genetics of yeast sporulation efficiency [17]. Not only does this data set provide us with a well-defined genetic system and a complex phenotype, but also it has been extensively analyzed by Cohen’s group, allowing us to compare ID results with the previous findings.

The dataset consists of 374 yeast progeny of a cross between two *Saccharomyces cerevisae* strains with very different sporulation efficiencies (3.5% and 99%). The sporulation efficiency of each strain was characterized by a real value between 0 and 1, which we binned into four integer values. We considered two binning strategies: optimal and uniform (see Methods and the discussion below). Each strain was genotyped at 225 markers distributed along the genome. Each marker is a binary variable since the strains from the cross are haploid and the alleles correspond to either parent A or parent B.

Initially we focused on single gene effects by calculating the conditional entropy 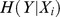 for the set of strains, where 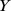 and 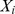 are the phenotype and a genetic marker (see Figure 1A). The three spikes in Figure 1A indicate the location of the single markers carrying significant information about the phenotypes. These are exactly the three quantitative trait loci (QTLs) previously identified in [17]. We also detect one of the two weaker QTLs, marker 7.17– the 17^th^ marker on the 7^th^ chromosome [17].

For genetic interaction analysis we calculate both the conditional phenotype entropy 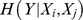 and 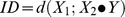 for all pairs of markers, 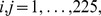 (Figures 1B–D). Recall that the conditional entropy 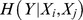 takes into account both single effects of individual markers and pair-wise interactions. Since there are three QTLs with strong marginal effects, plotting a heat map of 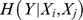 reveals three main stripes corresponding to these QTLs. Note that presence of some variation along the stripes indicates possible modifier interactions with markers that further decrease the phenotype entropy (Figure 1B). Note also that presence of individual spots of low entropy in a heat map of 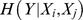 that are not part of a stripe would indicate possible synthetic interactions (see Figure 2). QTLs with strong marginal effects make detection of pairs of markers with small effect on phenotype using 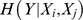 difficult. We use ID to disentangle the interaction signal from the single marker effects. Since 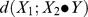 equals zero if either 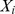 or 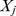 are independent from 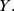 ID masks some of the single gene effects and helps identify two-gene effects. The ID heat map (Figure 1D) clearly shows that the effect of three major QTLs is reduced, leading to a much clearer characterization of genetic interactions. Figure 1C shows a slice across the ID heat map for a marker 7.9 that has a redundant effect with markers 7.8 and 7.11 and a synergetic effect with a marker 10.14.

We calculated the interaction distance after binning the phenotype using two different binning strategies, uniform and optimal (see Methods for more details on statistical tests), and evaluated the significance of interactions using four permutation tests with increasing stringency. Table 1 shows the most interesting pairs. Here we used the uniform binning of the phenotype and the most stringent statistical Test III, which accounts for possible marginal effects of markers. In order to explore the data further we used different binning strategies which reveal more significant pairs of markers (see Methods and Table S1 for more details).

**Table 1:** Interaction distances and the p-values from Test III for selected pairs of markers.

No.	Pair	ID	P-value
1	7.9, 10.14	0.20	<10^−7^
2	13.6, 10.14	0.043	4.5*10^−4^
3	13.6, 7.9	−0.014	0.39
4	10.14, 16.2	0.048	2.3*10^−4^
5	7.8, 12.13	0.060	0.016
6	7.8, 14.9	0.028	0.0053
7	10.14, 16.7	0.015	0.078
8	4.22, 10.14	0.044	4.9*10^−5^
9	9.7, 14.11	0.063	8*10^−7^
10	2.12, 4.23	0.04	3.5*10^−4^

The pairs were chosen to illustrate different aspects of practical use of interaction distance. Uniform binning of the phenotype was used to generate the table. An extended version of this is presented in Table S1.

Note that although the number of strains is not large enough to allow for consistent detection of subtle interactions (with significantly low p-values,) the interaction distance can be used as an effective filter for identifying the most interesting pairs that can be subjected for further analysis. We can compare our findings from Table 1 with a recent paper of Cohen’s group [18], in which ten additional QTLs with low effects on the sporulation were detected. This leads us to a set of candidate interactions with a likely effect on the phenotype. A detailed biological evaluation of these results is beyond the scope of this paper, but our goal is to demonstrate that methods based on ID can detect subtle effects that are most likely to be missed by other methods. These then can deliver biological hypotheses that are consistent with the most current biological knowledge. We present our candidate interactions in three groups: QTL interactions, modifier interactions, and synthetic interactions.

##### Interactions between QTLs

We first analyze interactions among the three markers with strong single effects on sporulation (7.9, 10.14, and 13.6). The top three pairs of Table 1 show the interaction distance for the corresponding pairs of QTLs. We found two strong interactions, between markers 7.9 and 10.14 and between 13.6 and 10.14, that have been previously detected [17], [19]. Note that the second pair illustrates the importance of different binnings: its p-value in case of uniform binning is 4.5×10^−5^ as shown in Table 1, while the optimal binning results in a more stringent p-value of 4×10^−7^ (see Table 2).

**Table 2:** Comparison of p-values of example pairs for different tests in the yeast example.

Pair	ID	Test 0	Test I	Test II	Test III
7.9, 10.14	0.20 (U)	<10^−7^	<10^−7^	<10^−7^	<10^−7^
13.6, 10.14	0.043 (U)	3.9*10^−5^	7.9*10^−5^	2.6*10^−4^	4.5*10^−4^
13.6, 7.9	−0.014 (U)	<10^−6^	<10^−6^	2.4*10^−4^	0.39
10.14, 16.2	0.048 (U)	4*10^−5^	2*10^−5^	2.6*10^−4^	2.3*10^−4^
7.8, 12.13	0.060 (U)	3*10^−6^	8*10^−5^	0.013	0.016
9.7, 14.11	0.063 (U)	<10^−6^	<10^−6^	<10^−6^	8*10^−7^
9.6, 14.11	0.044 (U)	6*10^−5^	6.6*10^−5^	7.8*10^−5^	8.3*10^−5^
7.9, 10.14	0.17 (O)	<10^−7^	<10^−7^	<10^−7^	<10^−7^
13.6, 10.14	0.073 (O)	<10^−6^	<10^−6^	<10^−6^	1*10^−6^
13.6, 7.9	−0.015 (O)	<10^−6^	<10^−6^	1.8*10^−4^	0.76
7.8, 14.9	0.046 (O)	2.8*10^−5^	3.4*10^−5^	6.8*10^−5^	7.2*10^−5^
1.2, 7.15	0.046 (O)	3.3*10^−5^	7.6*10^−5^	9.5*10^−5^	8.6*10^−5^
10.14, 16.7	0.045 (O)	4.4*10^−5^	5.3*10^−5^	7*10^−5^	7.1*10^−5^
2.12, 4.23	0.044 (O)	4.6*10^−5^	1.2*10^−4^	1.1*10^−4^	1.1*10^−4^

Symbols U and O stand for uniform and optimal binning respectively.

Although at first one might suspect some redundancy between markers 7.9 and 13.6, due to a negative ID value (−0.014), a careful statistical analysis indicates that this ID value is not significant (p-value = 0.39) and hence there is no interaction between 7.9 and 13.6.

##### Interactions between QTLs and their modifiers

We now analyze interactions, in which a marker with no single effect on the phenotype affects one of the three strong QTLs. Pairs 4–8 of Table 1 are some of the most interesting modifier interactions. For example, a possible interaction between marker 4.22 and QTL 10.14 (pair 8, Table 1) is particularly interesting because there are numerous genes related to sporulation efficiency located in the vicinity of 4.22 (chr. 4 - 1264114, www.yeastgenome.org). There are other markers that modify QTL 10.14, such as markers 16.2 and 16.7 (pairs 4 and 7, Table 1). Similarly, marker 14.9 interacts with 7.8, which is located about 55 Kbp from 7.9 corresponding to the QTL with the strongest effect on sporulation efficiency. It is unclear whether the interaction (14.9, 7.8) is related to the strong effect of 7.9 or is an interaction involving a small effect QTL reported in [18], which is located almost exactly between 7.8 and 7.9 (which are 55 Kbp apart).

##### Synthetic interactions

Finally, we analyze several interactions between markers that exhibit no marginal effects on phenotype. For example, the pair 9.7, 14.11 (pair 9, Table 1) has the second largest ID value in the current data set (only the interactions between 10.14 and 7.9 and their neighbors have higher ID scores.) The pair 2.12, 4.23 (pair 10, Table 1) is particularly interesting for two reasons: i) we have identified marker 4.22 as a modifier of a QTL 10.14, and ii) a marker located between 4.22 and 4.23 has been reported in [18] as a small effect QTL (the distance between 4.22 and 4.23 is about 98 Kbp and these markers are correlated). We thus conclude that this region of chromosome 4 has substantial genetic determinants of sporulation efficiency. All the dependencies involving 4.22 and 4.23 are especially interesting to us.

So far, we have been discussing pairs with the highest ID values. Since in this example we suggest to use ID as a filtering tool, we decided to extend our analysis to the top hundred pairs based on ID scores. We compared these pairs with findings of [18]. We found several possible candidates for interactions in this region (e.g., 4.15 with 15.10, 15.11 and 15.12; 4.16 with 15.1; 4.13 with 13.8). This suggests that this region of chromosome 4 may contain a cluster of small effect QTLs. This region of chromosome 4 exhibits a number of connections to chromosome 15 which has, to our knowledge, never been reported in the context of the sporulation efficiency.

Overall, the comparison with results from [18] shows that the interaction distance is a useful tool for detecting genetic interactions and QTLs with small effects on the phenotype, and can provide new biological hypotheses that may be tested in the future.

#### Analysis of data from a mouse cross

We next apply ID to a more complex biological system – a mouse cross. There are several sources of increased complexity in the mouse system (as compared to yeast): i) phenotype data is more subtle and therefore noisier and harder to bin, ii) data sets are typically smaller (due to a limited number of progeny), and iii) the genotype data is three-valued (unlike the two valued haploid yeast genotypes) – QTLs can be homozygous (AA and BB) or heterozygous (AB). All these differences can cause a lack of statistical power when searching for interactions between small effect QTLs.

The data set, kindly provided by Jake Lusis [20], [21], consists of 334 mouse progeny of an F2 intercross derived from the inbred strains B6 and C3H on an apolipoprotein E null background. Several phenotypes associated with metabolic syndrome of each strain were characterized. In this example we analyzed the phenotypes LDL cholesterol level and body weight. Due to the sex difference effects on weight, we considered male and female weight separately. Thus four cases were analyzed: LDL, W (weight), MW (male weight) and FW (female weight). All the phenotypes were uniformly binned in two bins with the median as the boundary. Each mouse strain was genotyped at 1285 markers (SNPs), where each marker is a variable that takes three values (AA, BB, AB).

As for the yeast analysis, we start with the analysis of the conditional phenotype entropy 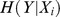 to detect dependence of the phenotype on a single genetic marker for all mice, and separate groups by sex. Figure 2A,D,G illustrates this entropy applied to weight. Notice that due to a large sex difference of weight, all the markers from the sex chromosome 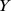 are detected as “QTLs” when we considered the entire population of mice (Figure 2A). To analyze the compound influence of two markers on the phenotype we compute 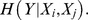 A heat map of 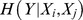 (Figures 2B, E, H) shows a structure of stripes corresponding to QTLs with major effect on the phenotype. Notice that the stripes are not uniform and the variation in the intensity of the stripe corresponds to possible modifier interactions with these QTLs. Moreover, the heat map reveals numerous spots separate from the stripes that correspond to possible synthetic interactions. Since 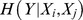 detects a combination of both single gene effects and pair-wise interactions, we use ID to extract genetic interactions.

We computed ID values for all pairs of markers and selected the top pairs based on p-values and the missing data threshold (see Methods for details). Although these criteria are strict, resulting in selection of a very small subset of candidate pairs, they lead to interesting observations and frame useful hypotheses. Table 3 shows several interesting interaction candidates for LDL, FW, and MW (see also Table S2, Table S3 and Table S4 for more details). Notice that p-values are not as low as in the yeast sporulation example above. This is likely due to a smaller number of samples and higher numbers of possible QTL states (the marker density is substantially higher than in yeast.) Nevertheless, we argue that ID can be used as an effective filter for finding pairs of markers that may be interesting for further study. One major difference we noted between the mouse and yeast examples is that in the mouse case almost all top scoring pairs are synthetic interactions. The only interaction between two strong QTLs was detected for the LDL phenotype. There are also only one or two modifier interactions in each phenotype (see Table 3).

**Table 3:** Interaction distances and p-values from Test III for selected pairs of markers for mouse phenotypes.

Phenotype	Pair	ID	P-value
LDL	454, 388	0.0398	1.2*10^−4^
LDL	646, 591	0.0402	1.5*10^−5^
LDL	691, 269	0.047	1*10^−5^
LDL	**891**, 542	0.044	1.5*10^−5^
LDL	934(M), 96	0.0451	4*10^−5^
LDL	966, **878**	0.0412	7*10^−5^
Weight, fem.	773, **68**	0.082	5.1*10^−4^
Weight, male	135, 57(L)	0.083	1*10^−4^
Weight, male	148, 57(L)	0.097	1.2*10^−5^
Weight, male	746, 362	0.081	2.4*10^−4^
Weight, male	876(L), 566	0.088	1.4*10^−4^
Weight, male	890(L), 367	0.087	5.6*10^−5^
Weight, male	388, 281	0.083	2.1*10^−4^
Weight, male	791, 269	0.084	4.3*10^−4^
Weight, male	1021, 84(F)	0.081	3*10^−4^

The underlined markers are QTLs with significant effect on a corresponding phenotype: the bold markers are the strongest QTLs (p-value<∼0.0001), while the other underlined markers are QTLs with smaller effects (p-value <0.001 in simple permutation test). The indicators (M), (F), or (L) next to some markers mean that the marker has an effect on the male, or female weight, or LDL respectively.

Researchers from the Lusis lab characterized this mouse cross for 27 different phenotypes. In particular, they included fat mass [20] and arterial lesion size [21], and detected numerous QTLs with strong marginal effects. We compared these QTLs with our ID results. Note that although we considered different phenotypes, they are measurements of the same biological system under the same conditions. Moreover, we observed a very strong correlation between fat mass and weight. Therefore, it is interesting to note that some of the same QTLs appear in different phenotype contexts.

ID detects locus 388 interacting with loci 454 in LDL and 281 in MW (see Table 3). Although neither of these three markers have single gene effects on MW or LDL level, marker 281 has been recognized as a QTL strongly affecting fat mass [20]. We conclude that locus 388 is a strong candidate for further investigation relevant to LDL level, body weight and fat mass. Other results of potential interest include marker 269 that interacts with 791 in MW and 691 in LDL level, and marker 57, a QTL affecting LDL level, that interacts with markers 135 and 148 in MW. These loci have not been noted in previous work [20], [21] and such double interaction signals in various phenotypes are highly suggestive.

Prior results show that locus 361 is recognized as a QTL strongly affecting the arterial plaque lesion size [20], [21]. Using ID we detected an interaction between loci 362 and 746 in MW (there is also a weaker interaction between 361 and 746). Moreover, marker 363 has a clear effect on male weight. This suggests that region [361–363] of chromosome 4 (the distance between markers 361 and 363 is about 5 Mbp) is important in the context of phenotypes of our interest. Interactions of 746 with markers from this region make it potentially interesting for further study, which can shed some light on the genetic effects caused by these regions of the genome. Several similar ID results include interactions in FW between markers 773 and 68–69 (two highly correlated markers that are about 1.5 Mbp apart) corresponding to the strongest QTL of the fat mass [20], and an interaction in LDL between markers 96 and 934, which is the QTL with the strongest marginal effect on the MW phenotype. For more examples see Tables S2–S4.

Markers detected in the previous work appear prominently in the set of interactions for weight and LDL detected using ID. In other words, our ID-based analysis provides evidence that strong effect QTLs in one phenotype can have significant effects on other phenotypes by participating in additional interactions. Moreover, these examples demonstrate that results provided by the analysis based on the information theory measure ID can lead to biological hypotheses for further investigation.

#### Analysis of simulated human data

We increase further the complexity of the system considered now and apply ID analysis to human data. Our aim here is to carefully consider different challenging properties of human data and explore the modifications required for ID to handle them. The primary challenges are the variations in allele frequency, and the high levels of diversity encountered. To have close control of the data parameters, we use simulated data obtained from several well-established models representing pairwise interactions. For comparison, we simultaneously apply interaction information (II), a non-normalized measure previously used for genetic data filtering [9], [10]. To enable the reader to reproduce these results and apply presented tools to their data we added Matlab scripts as a supporting material file (Code S1).

Most human genetic studies are medically motivated. It is increasingly evident that most disease phenotypes involve complex genetic interactions [1], [22], despite the predominance of GWAS, limited to single-gene effects and additive pair-wise interactions. The relatively small size of these data sets and difficult sampling issues (case-control ratios, population structures) render most approaches to identify multi-gene effects, extremely difficult. Moreover, as opposed to genotypes from intercrosses of model organisms, human genetic data, mostly from populations of unrelated individuals, consists of SNPs with non-uniform, and highly heterogeneous allele frequencies. It is well known that many disease-related genetic variants have low minor allele frequencies (MAFs): the range varies between 0.5% and 50% (see [23]–[25] and Table 1 from [22]). For example, MAFs of three causal variants of Crohn’s disease detected by GWAS are below 5% (4.1% 1.9% and 1.5%), and MAFs of variants related to sick sinus syndrome and those related to ovarian cancer are reportedly below 1%. In the analysis of genetic interactions, or QTLs, in human populations, SNPs with divergent and often times low MAFs are problematic, since they make detection and comparison of signals from SNPs challenging. Note also that the MAF is calculated from the original population. However, when we construct a case–control data set, where the number of cases is usually about 50%, the actual allele frequency may be considerably different.

**Figure 3:**
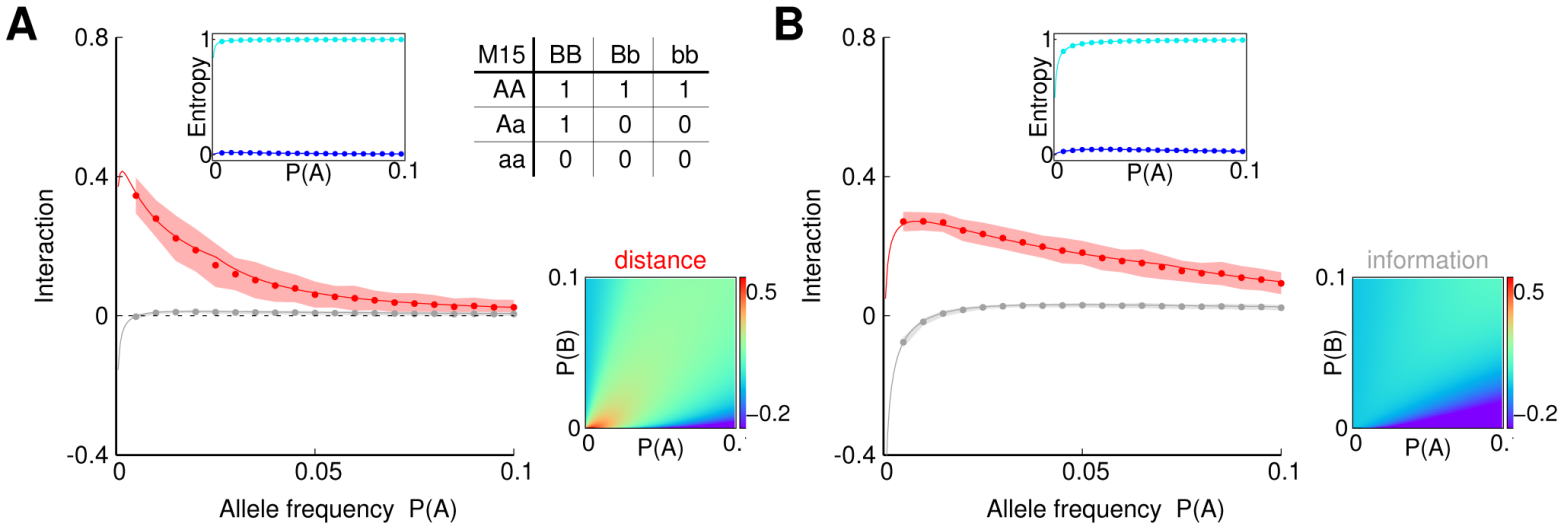
Detection of SNP interactions in a human disease model. Each main panel shows interaction distance (red) and interaction information (grey) computed on simulated data from a human disease model M15 of the interaction between SNPs A and B defined in the table. Solid lines describe analytical expectation values, dots show average values obtained from simulations and shadowed bands describe corresponding standard deviations (see Methods for more details). The upper sub-panels show the conditional entropy of phenotype given SNP A (blue) and B (cyan), respectively. The entropy illustrates strength of marginal effect of a given SNP Minor allele frequencies of SNP B were fixed to 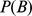  = 1% in panel (A) and 2.5% in panel (B) Lower sub-panels show the effect of changing the value of 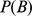. More precisely, the lower sub-panel on the left shows expected values of the interaction distance, and on the right – of the interaction information as functions of different values of 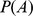 and 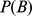.

**Figure 4:**
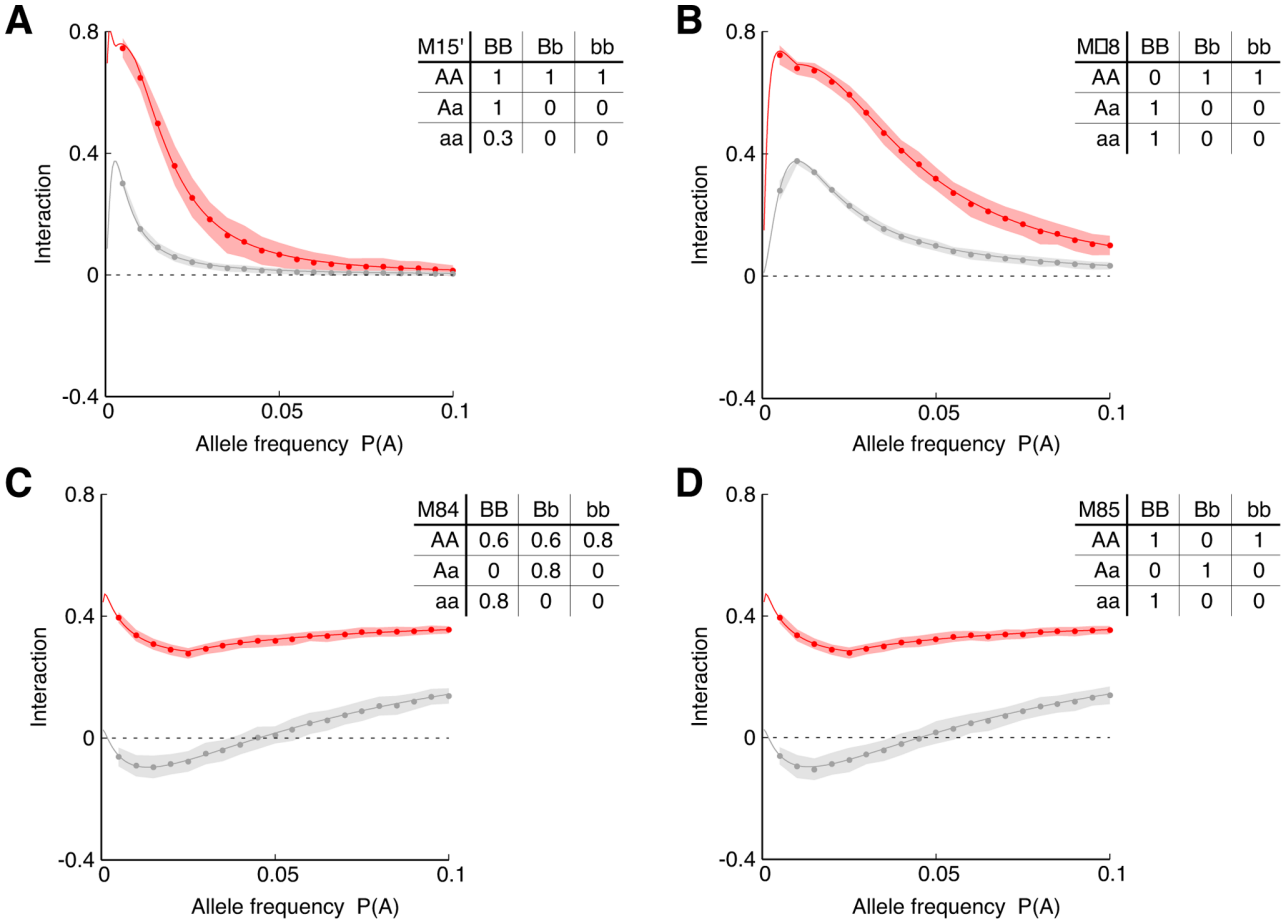
Detection of SNP interactions in further disease models. Additional simulations showing performance of the ID (red) and II (grey) for various models. To mimic a scenario in which the disease can be caused by other factors (e.g., other mutations, environmental factors) we added noise to some of the models, which take form of non-binary penetrance tables.

To illustrate and benchmark the use of ID and II, we use several well-established models for pairwise genetic interactions [9], [26]. Specifically, we focus on disease models M15, M78, and M84 [26] and their modifications (Figure 3 and Figure 4). These models represent various ways the allele combinations of two subject SNPs can affect the phenotype. The two SNPs were assumed to be in linkage equilibrium. M15 models a modifying interaction where only one marker alone has a marginal effect on the phenotype. Models M84 and M85 illustrate that even if both markers have marginal effects, detecting an interaction between them may be difficult. The penetrance functions in the matrices in Figures 3 and 4 (most of which are binary) represent the probability of a disease phenotype occurring for a given combination of alleles. In the case of the models M15, M78, and M84, the probabilities are zero or one, reflecting zero or full penetrance. In the modified models we reduced the probability values corresponding to more variable penetrance effects. We consider effect of different penetrance values in more detail below. Figure 3 presents a detailed analysis of the expected values of II and ID, and other auxiliary measures such as phenotype entropy, for a population sampled from model M15 with equal number of cases and controls (see Methods), which is typical for cohorts used in GWAS. For a more detailed analysis, we simulated the genetic scenarios by generating cohorts with 500 cases and 500 controls using fixed values of one MAF, 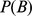 in our case, and varying 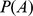. Results for other models are summarized in Figure 4. In contrast to the mouse and yeast applications shown in the previous sections, here our goal is to see if the measures, ID and II, are able to detect specific input relationships with different parameters.

For all examples shown, the interaction distance has higher values than interaction information (the standard deviation bars indicate significance information). This difference, however, can be attributed to the re-scaling due to the normalization (see Methods section for a detailed discussion of the effect of normalization). More interesting is the fact that although the interaction information is either negative or close to zero when 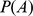 is low (low MAF) for M15, M84 and M85 models, the interaction distance is positive. This suggests that ID is able to detect interactions where II fails, especially for small MAFs. To understand this behavior, let us look at the composition of the case/control data set.

For example, consider model M15 with MAFs of A and B equal to 0.5% and 2.5% respectively. In this case the average values of 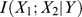 and 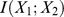 are 0.36 and 0.43 respectively, and consequently the interaction information is 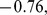 which indicates that 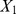 and 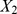 are redundant and have no interaction. Note however that variables 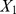 and 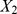 are supposed to be independent and the unconditional mutual information is expected to be zero. This would be the case if the data set reflected the actual population but the presence of sampling bias towards affected individuals creates a false correlation among the causal variables. The interaction distance is more robust in this situation. Indeed, the normalized mutual information values are 0.58 and 0.32 for the conditional and unconditional cases respectively, resulting in ID value of about 0.26, which correctly indicates a presence of interaction between 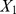 and 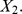


Recall from Equation 1 that II is a difference between conditional and unconditional mutual information. Mutual information is a measure that depends on the entropies of the two variables and it is never higher than the minimum of the two entropies. Therefore, taking the difference between unnormalized mutual information values for variables with very different entropies can lead to negative values of II where positive values are expected as shown in Figures 3 and 4. On the other hand, ID has a normalization formula that boosts the value of the conditional mutual information and suppresses the unconditional one, providing a corrective effect.

When the values of 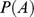 increase, both measures tend to zero, which is expected since the number of causal variants A outnumbers the causal variants B. In consequence, the effect of A masks the effect of B.

Figure 3 and Figure 4 illustrates that both methods are potentially suitable for detecting interactions of SNPs with low MAF values. However, the interaction distance is often a more preferable choice over the interaction information especially in the extreme cases of human SNP data.

To examine the effect of different levels of penetrance on interaction detection we add an extra tunable parameter to a disease model: all the values of the “risk” genotypes (all the 1′s) in the disease penetrance table are replaced with this parameter, thus controlling how often the risk genotypes result in a disease. We studied the behavior of ID for various values of the penetrance parameter across all the models and found no significant effect of the penetrance on ID. Indeed, since all the risk genotypes have the same penetrance, the genetic composition of all the cases in the case-control data set stays the same when we change the penetrance parameter from 1 to a lower value. Similarly, the genetic composition of all the controls also stays the same (or almost the same) when we change the penetrance. Note that with the penetrance parameter less than 1, a number of individuals with the risk genotype will not have a disease and will be added to the set of controls. However the number of such individuals is very small due to low MAFs of the risk genotypes.

One should be aware that the majority of pairs of SNPs has no effect on the phenotype, and thus, they can contribute to noise that will have an impact on the results provided by ID (or by any other measure). Let us now briefly consider the effect of this background noise. We investigate this by generating background distributions (see Methods section for more details) for various allele frequencies where both SNPs are independent and have no marginal or pairwise effect on the phenotype. We generated 20 million pairs of SNPs with various MAFs ranging from one to fifty percent, one million for fixed frequencies. The maximal observed value of the ID score was 0.019, the average was about 0.002. This seems to be significantly different from the ID scores observed in many cases illustrated in Figures 3 and 4, where the values of ID are often above 0.1. One must realize, however, that presence of various types of noise, like genotyping or measurement noise for example, plus an enormously large number of pairs may lead to difficulty in obtaining significant results. Therefore, we argue that the best way to using ID (and other measures based on information theory) is as a filtration tool. We demonstrated this approach in the mouse example, where we provided lists of candidate pairs of interacting markers and compared them to previous findings which led to formulation of new hypotheses.

In the current paper we do not provide any analysis of a false positive rate or ratio of false to true positives. This follows from our philosophy of how ID (or II) should be applied in practice, which is, as stated above, a filtration approach. We have to remember that in order to analyze false positive rate, one has to make a final decision whether a pair of markers interacts or not. While a filtration approach is based on ID threshold, which is not too stringent, a final decision should be based on a permutation test. But this is strongly dependent on the context of available data. For instance, if we have only 200 markers which results in 20 000 pairs an ID score with p-value as high as 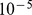 would be considered as indicating an interaction. Even pairs with p-values of 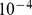 can be considered as significant, especially, if we detect high number of those. For example, if we see 20 such pairs we can hypothesize that at least some of them are actual interactions. On the other hand, in human case-control studies with 500 000 SNPs a p-value of 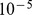 cannot be considered as significant. Here, filtration seems to be a more suitable approach.

So far we have been assuming independence of alleles from different loci. Let us now briefly discuss a case when two SNPs are not independent and are in so called Linkage Disequilibrium (LD). In other words this means that alleles of two different loci are correlated. LD between markers makes proper permutation testing of interaction between these markers challenging. Although both markers in LD have marginal effects on the phenotype, Test III is not applicable, since both markers are correlated and the randomization performed during the testing procedure affects that correlation. Hence, formally speaking, we cannot make any statement about potential interaction between such markers based on this test.

We analyzed the same models as depicted in Figures 3 and 4 with different values of LD (see Methods for more technical details). The general observation is that presence of LD lowers values of both analyzed measures, ID and II. We also observed that II scores were lower than ID scores in all analyzed examples. We also analyzed models in which only one marker has a causal effect on the phenotype, and the other is just correlated with the previous one. In such a case, values of ID and II are either zero or below zero in cases of stronger LD. In fact, this is an expected behavior since in such a case the two markers contain redundant information about the phenotype. Thus, negative values of ID and II. We can observe such a situation in Figure 1C where we see a negative peak of ID values for a marker with strong effect on the sporulation an its neighbors.

We conclude that although in many cases LD makes detection of potential interactions more difficult, it does not lead to false positives since non-interacting markers that are linked result in low ID scores and are not selected for further investigation during the filtration step. The main scope of this paper is to examine interactions where at least one marker has no marginal effect, which does not happen in case of markers in LD. A detailed analysis of the influence of LD on detection of interactions between markers with marginal effects will be a topic of our future work. The problem of constructing a permutation test seems to be especially interesting.

**Figure 5:**
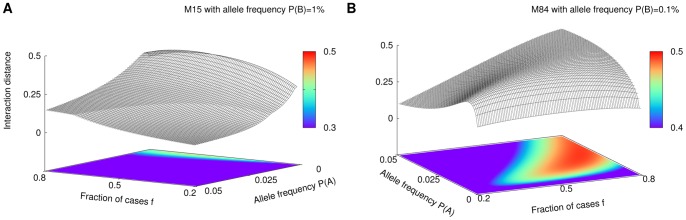
Relationship between the case-to-control ratio and ID. Among other factors, optimal detection of synergistic effects depends on the case-to-control ratio *f* of the study. Panels (A,B) show the dependency of ID values on allele frequencies and ratio *f* for models M15 (A) and M84 (B).

The model analysis shown in Figures 3 and 4 allows us to analyze the effect of the ratio of cases-to-controls in a clinical data set for each of the models and the different MAFs. We observe that, given a particular model, both ID and II are significantly dependent on the model parameters, and that there are optimal values of the case-to-control ratios (see Figure 5). This suggests that if the MAF or other parameters can be estimated as in validation studies, the choice of this ratio can be optimized.

## Discussion

Information theory approaches to data analysis can be used to good effect in addressing genetics problem of several kinds. The information measure called interaction information has been recently used for finding associations between two SNPs in human data [9], [10]. These results are promising, but we demonstrate here that this measure can be improved upon for specific types of data, such as human population data with wide range of minor allele frequencies. When allele frequencies are highly skewed (very low MAFs) the entropies at these sites can be very low. This is especially true for the conditional entropies. As a result, the unnormalized interaction information is close to zero which, particularly in the presence of noise, can prevent the detection of interactions. We have shown here that the information theoretic measure, interaction distance, a normalized measure defined by analogy to information distance and interaction information by a recursion relation, can be a very sensitive and useful measure. On the other hand, it should be said that SNPs with very low MAFs may also be problematic because of limitations of current genotyping technologies. Methods like ID cannot help if such problems occur.

We have applied our measure to both yeast and mouse cross data sets, with equal allele frequencies across the genome, which works reasonably well. It is particularly useful, however, in application to human data. We show that for several models the normalized measure, ID, can be particularly useful for low MAF cases by providing a compensatory effect for large differences in the entropies of the marker variables. The yeast and mouse examples demonstrate a powerful use of the interaction distance as a tool for choosing pairs and markers for further investigation. Even though we developed strict methodology for statistical testing of the ID and II scores, the number of strains/samples available may often be insufficient to provide p-values in a useful range. We argue, therefore, that the results provided by the ID analysis should be compared with results related to similar or related phenotypes. In both, yeast and mouse, cases we were able to identify QTLs that are very likely to be involved in several of the phenotypes analyzed. We observed that very often a QTL having strong effect on one phenotype may have a small effect on another, related phenotype, often due to interactions. This kind of analysis can result in biological hypotheses and potentially better understanding of the architecture of genotype–phenotype relationships, and is an important and promising direction.

We will be applying ID analysis to more sophisticated models and to real data sets. Since, the genetic data typically consists of hundreds of thousands SNPs, the problem is computationally challenging but our test cases demonstrate that it is tractable. The analysis is completely parallelizable and with proper parallelization we can perform all computations on a million SNPs data set within a relatively short time on multi-core desktop machines. Of course, this refers to calculations of the ID only. The permutation tests are computationally expensive and can usually be performed only for selected sets of pairs.

Finally, it is important to point out that the extension of genetic analysis here to the three-variable case, while at the edge of current capability is just the first step. We have developed a general, multi-variable formulation based on information theory, to be published elsewhere, that will allow us to perform analyses of the interaction of a larger number of markers, and of more complex interactions.

## Methods

### Different Normalizations

Let us briefly discuss the normalization issues. The information distance (Equation 2) is normalized by the higher of the two entropies. This is done to guarantee the metric properties. Another possibility is to normalize by the minimum entropy [27], since the mutual information is bounded by the smaller value of the two entropies. There is a practical difference between these two normalizations, however.

Since
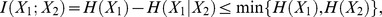



let us assume that 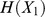 is the smaller of the two entropies. Let us suppose now that the mutual information normalized by 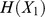 equals one, which means that the conditional entropy 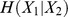 is zero. Thus, there must be a functional relationship, , meaning that if we know 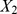 then we also know 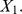 On the other hand, it does not mean that the knowledge of 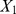 gives us full knowledge of 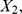 since 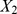 could be a multi-valued function of 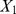, for example.

When the mutual information is normalized by 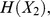 the higher entropy, implies existence of a two-way functional relationship, 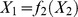 and 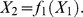 We choose this normalization in the construction of the normalized information distance (see Equation 2) to guarantee that the distance is zero if and only if the two variables are equivalent. If we normalize by the minimum, it may happen that the distance is zero but one of the variables (with the higher entropy) carries more information.

It is unclear at this stage which type of normalization would be preferable in our biological applications. We observed that normalization plays a marginal role in case of mouse and yeast data: II and ID produce almost equal results because of the homogeneity of the variables (50% MAFs). The variables representing genetic markers in these types of experiments have similar entropies, thus, the effect of normalization is negligible. The statistical significance of the observed results is also the same. On the other hand, there is a significant difference in performance between II and ID in case of the human models where the MAFs are not uniform.

### Properties of the Interaction Distance

In [15] a theorem describing the most important properties of the interaction information was proved. It states that.

**Theorem 1.** If 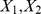 and 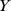 are finite random variables, then:

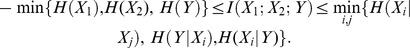

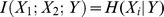
 if and only if 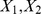 are independent and 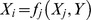 where 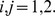

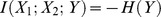
 if and only if 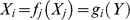 where 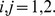

The first property shows the range of II scores. The next two properties suggest the interpretation of the II values: positive II scores suggest synergistic effects while negative scores indicate redundancy. The functional relation in the second property means that the knowledge about the states of two variables results in the knowledge about the state of the third one, on one hand. On the other hand, any two variables are independent. The relation presented in the third point represents a situation when 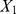 and 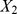 contain redundant information about 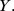 Moreover, in such a case 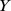 determines the other two variables, and by knowing state of 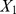 we can extrapolate to the state of 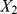. Of course, these two relationships are extreme cases of functional relations between variables, and in practice we observe probabilistic versions of these cases. The randomness observed in real data may be either a result of noise (e.g., experimental, environmental) or a result of the fundamentally probabilistic nature of the process described by the model.

We now present the counterpart of these results that establish the basic properties of the interaction distance. Large parts of the proof remain the same as for II. Remember that we consider only finite random variables, i.e., maps defined on a probability space with finite state space.

**Theorem 2.** The interaction distance has the following three properties:

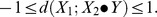

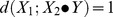
 if and only if 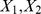 are independent and 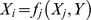 where 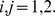

3. If a sequence 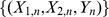 converges in law to 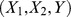 such that 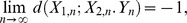 then 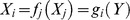 where 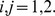 We need to assume, moreover, that all the variables 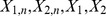 are defined over the same alphabet (the set of possible states which the variables can take with a non-zero probability).

**Proof.** 1. By definition, the ID is a difference between two measures normalized to the interval 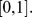


2. Suppose 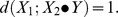 This implies 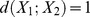 and 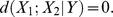 The first equality implies that 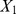 and 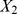 are independent. From the second equality, it follows that 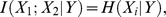 which leads to 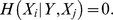 This implies 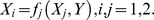.

The proof of the converse statement is trivial. Note that 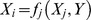 does not contradict the independence of 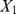 and 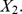 This is the case when, for example, 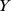 is the sum of 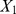 and 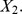 In such an example both functional relationships occur: 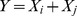 and 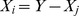.

3. The convergence of interaction distances implies that (a) the unconditional distance converges to zero and (b) the conditional distance converges to one.

- Since the unconditional distance converges to zero, 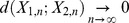, we have 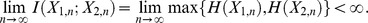

  This implies that 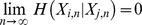 (this follows directly from the representation of mutual information as a difference between conditional and unconditional entropies). Since we consider finite random variables defined over the same alphabet, this implies that 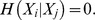 Equivalently, 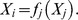

- Since the conditional distance converges to 1, the ratio 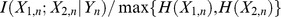 converges to zero as well. Given that the denominator is bounded, the conditional mutual information converges to zero which leads to the statement of property 3 of Theorem 2 as presented in the proof of Theorem 1 in [26].

According to property 1 of Theorem 2, ID is normalized to the range from −1 to 1, as opposed to II that ranges between 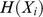 and 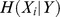 from the original (II) version. This gives ID an advantage over non-normalized II: when the values of entropy are low, even highly deterministic interactions have the interaction information values close to zero. As a consequence, such interactions could become indistinguishable from the noise. This is problematic in data sets composed of variables with various entropies. For example, while both II and ID give similar result on the yeast and mouse data, where entropies of all the variables are very similar, ID outperforms II on the set of simulated human SNPs interactions, where entropy of corresponding variables takes on a wide range of possible values.

This can be further illustrated by an example where both 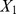 and 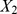 are independent binary variables and 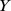 is a modulo 2 sum of 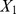 and 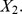 Property 2 of both theorems applies in this case. When, 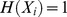, i.e., 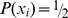, both II and ID equal one. However, if we change the prior distributions of 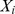, and decrease the entropy, then the value of II also decreases while the value of ID remains equal to one.

According to property 3 of Theorem 2, ID values close to −1 indicate that the value of 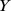 determines values of both 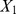 and 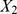, or in other words, 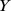 has a causal effect on 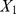 and 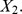 Following the proof of the theorem, for ID to be close to −1, the unconditional distance 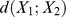 must be close to zero, indicating a functional relationship between 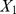 and 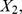 and the conditional distance must be close to one, indicating that 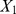 and 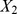 become conditionally independent given 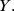 Note, that in this paper we focus only on the positive values of II and ID, and the difference between these measures when they are negative is a direction for future research.

### Permutation Tests and Computation of p-values

The current section presents detailed description of the permutation tests used for calculating the p-values of the interaction distance between two markers. We can make a rough estimate of p-values by generating 1 million pairs of random markers with no missing alleles and the same allele frequency as in the original data. Computing ID for these 1 million pairs and the phenotype, which remains untouched, generates a background distribution used to test the significance of the original ID values. We refer to this statistical testing as *Test 0*. Note that Test 0 gives only a rough estimate of p-values since it does not account for missing data. Moreover, the Test 0 background distribution is generated under the null hypothesis that i) both markers have no effect on the phenotype and ii) there is no interaction between the markers. Therefore, formally speaking, a rejection of such a hypothesis does not imply the presence of an interaction between markers. For example, the null hypothesis may be rejected if one of the markers has an effect on the phenotype. To increase the accuracy of the significance testing we propose three permutation tests (*Test I-III*) with different null hypotheses.

The tests are performed for each pair of markers separately. Let us suppose that 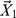 and are vectors of alleles representing the markers of our interest, and 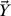 is a vector representing the phenotype binned into 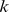 classes. The length of all these vectors is 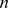, where 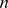 is the number of samples, e.g., yeast strains, mice, patients. Formally speaking, the data can be written in a form of 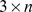 matrix: 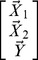.

The background distribution is obtained by generating vectors 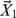 and 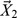 randomly from a prior probability distribution 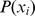. In some biological applications these distributions are known. For example, in yeast we have two equally distributed alleles and in mice we have three combinations of alleles distributed with probabilities 0.5, 0.25 and 0.25. On the other hand, we are not able to provide a universal background distribution for the human analysis, since the MAFs vary from 0.5% up to 50%.

Test I is performed by randomizing 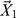 and 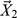. From here on we say that a vector is randomized if its values are randomly permuted. Obviously, this procedure preserves the number of missing values and the proportion of different alleles while randomizing the effect on the phenotype and the interaction between markers. Therefore, the null hypotheses of Test I and Test 0 are the same, however Test I accounts for the missing data. From a formal point of view, this should be used for testing significance of interactions between markers with no individual effects on the phenotype. In the case when one of the markers in a pair has an effect on the phenotype and the other does not, we should use Test II that randomizes only the vector corresponding to the marker with no effect and preserves the other two vectors (the phenotype and the other marker). The null hypothesis of Test II is that i) 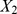 has no effect on the phenotype, ii)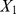 has an effect (that can be measured by a reduction of 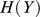 to 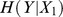 ) and iii) there is no interaction between 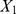 and 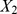.

The most difficult is the case of two markers having marginal effects. Test III is designed to preserve the effects of the markers but randomize a possible interaction between them, so the null hypothesis is that two markers with effects 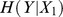 and 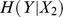 do not interact. To test such a hypothesis we have to randomize markers in such a way that the conditional entropies are not changed. Without loss of generality, let us suppose that vector 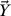 is ordered in the following way: , where 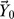 is a zero vector, 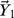 is a vector of ones, and so on. We also assume that 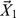 and are ordered accordingly. Consequently, the matrix 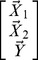 can be rewritten as 
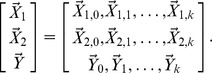



During Test III, we randomize each sub-vector 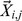 separately, which does not change values of the conditional entropies. Therefore, the null hypothesis of Test III is that there is no interaction between 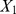 and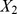. The advantage of Test III is that this null hypothesis fits to all three cases: no marginal effects on the phenotype, a marginal effect of only one marker, and marginal effects of both markers.

### Details of the Analysis of Yeast Data

Since the approximation of information theory measures, such as mutual information, for continuous variables may be difficult and requires large number of samples, we binned the phenotype into four bins. In the current work we use two binning strategies: i) a uniform binning with 0.25, 0.5 and 0.75 quantiles as thresholds and ii) a so-called optimal binning proposed in [28]. The optimal binning strategy uses hierarchical clustering and determines the coarseness (the number of clusters) that maximizes the interaction information for pairs of markers and the binned phenotype. The number of bins determined by the optimal strategy is four and the number of data points in each bin is 120, 89, 95 and 70. Note that the use of interaction information for binning motivated us to further investigate this subject and led to the concept of ID. Although the difference between these two binning strategies is not very large, it still results in some differences between the pairs with the highest ID scores. Table 2 shows the pairs of markers with the highest ID values calculated with both binning strategies. Moreover, Table 2 provides p-values estimated using four different significance testing, i.e., Tests 0-III.

We are aware that binning, discretization of continuous variables, leads to loss of information. Nevertheless, the information content of the binned variables is sufficient to detect the signal of interacting pairs of markers. In practice, we suggest exploring various binning strategies with different number of bins. In the presented yeast example, the number of bins (four) was “optimal” both in terms of statistical power and number of discovered candidate pairs. Since we are mainly interested in applications to human data where phenotypes are often discreet (case-control studies), we do not analyze approaches based on approximating mutual information and entropy from continuous data such as kernel based approximations.

### Details of the Analysis of Mouse Data

**Figure 6:**
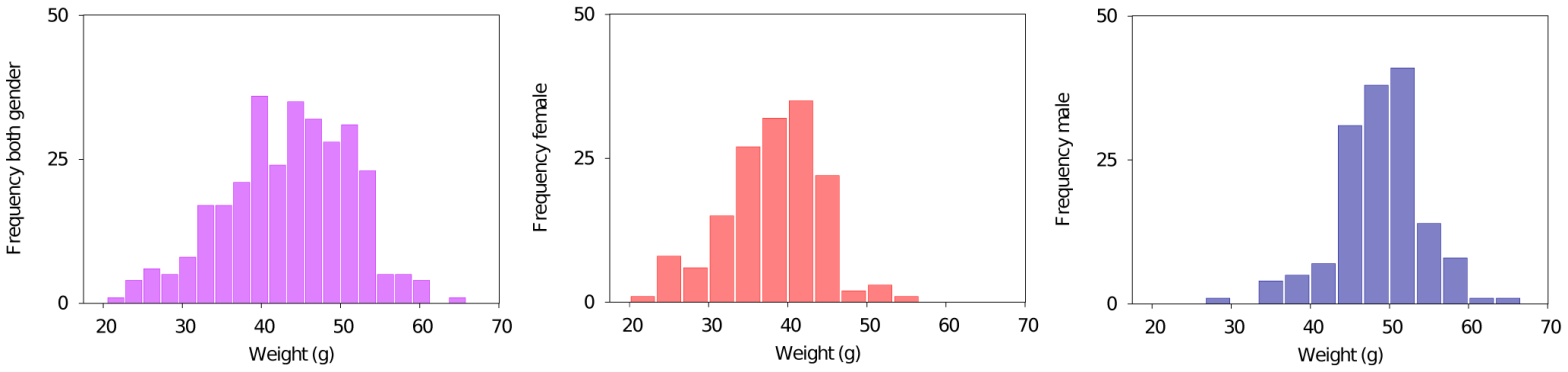
Distribution of mice phenotype. Distribution of mice weight exhibits clear sex dependence. Male mice (blue) are significantly heavier than female (red). The merged unisexual distribution (magenta) exhibits larger variation.

We applied ID to two mouse phenotypes: the LDL cholesterol level and weight. We observed that male weight is considerably more different than female weight (see histograms in Figure 6). Consequently, we considered the mouse weight for both males and females together and for each gender separately, whereas LDL cholesterol level was considered only for both genders together. In all four studies, a phenotype was binned uniformly into two integers according to the phenotype’s median. This is probably the simplest binning strategy but the number of mice was too small to consider any more sophisticated approaches.

Note that sex difference of the weight affects the binning and consequently the downstream interaction analysis. Indeed the ID analysis performed on the entire population identifies only sex specific markers. Therefore, we conduct a separate analysis for male and female weight.

We performed the ID analysis for all the pairs of markers for LDL (both genders) and for weight (males and females individually) and then estimated their Test 0 p-values. Tables S2–S4 show pairs with p-values below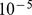. We also removed the pairs with more than 10 missing values. Significance of the selected pairs was also tested using Test I-III.

Note that whenever we observed blocks of consecutive markers interacting with each other, we selected the pair with the highest interaction distance as a representative of similar interactions. For example, the pair (691, 269) in LDL represents a group of interactions between markers 691–693 (distance between 691 and 693 is about 3 Mb, and the mutual information between them about 0.83) and 267–273 (distance between 267 and 273 is about 11 Mb, and the mutual information is about 0.84), since (691, 269) has the highest ID score among all of these pairs. Note that although the distance between markers within a block can be large (up to 11 Mbp), these markers are strongly correlated. We might mix two or more biological interactions together, however we are not able to distinguish these interactions in a block of such highly correlated markers.

### Computing ID and II for Clinical Data Sets

In order to simulate a clinical cohort data with the same number of cases and controls, we generate independently two versions of each SNP according to the probabilities 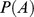 and 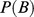. This gives us a genotype of an individual, who is then classified into either a case or a control according to a penetrance function. This procedure is repeated until we obtain a desired number of cases and controls. Figure 3 shows the results averaged over 100 such cohorts. If SNPs are in LD, then probabilities of observing particular combinations of alleles are modified by normalized deviation from equilibrium, 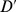, defined as in [29].

In order to estimate the expected values of II and ID we calculate the expected frequencies of each genotype within a healthy and affected population (i.e., within cases and controls). For example, the probability of observing genotype AABB given an affected individual can be written as:
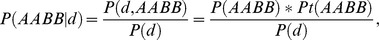
where 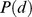 is a frequency of cases in the data set, which usually equals to 0.5 in the case-control type of study, and 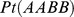 stands for a value of the penetrance function for the input 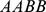. Finally, if the alleles are inherited independently (no LD), we have:



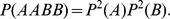



If the two SNPs are in LD, then the above formula needs to be written as:
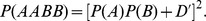



In case of allele configuration 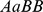 the probability 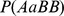 would be equal to 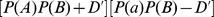.

Obviously, with the growing number of samples, the observed frequencies are getting closer to the conditional probabilities calculated as presented above. Therefore, given the allele frequencies, we use these theoretical values to approximate the expected values of ID and II for the case-control data models. Figure 3, illustrating the interaction analysis results computed on 500 cases and 500 controls, shows that the measured and predicted expectation values of ID and II are practically the same. All simulations were performed in Matlab; the source code can be found in a supporting material file (Code S1).

## Supporting Information

Table S1 Comparison of p-values of example pairs for different tests and both binnings in the yeast example.
(DOC)

Table S2 Comparison of p-values of example pairs for different tests in mouse LDL phenotype.
(DOC)

Table S3 Comparison of p-values of example pairs for different tests in female weight phenotype.
(DOC)

Table S4 Comparison of p-values of example pairs for different tests in male weight phenotype.
(DOC)

Table S5 Coordinates of selected markers from the mouse example.
(DOC)

Code S1 This file contains Matlab scripts that will enable readers to reproduce results presented in the paper and apply presented tools to their data.
(ZIP)
