# Supplementary material for: Discovering Pair-Wise Genetic Interactions: An Information Theory-Based Approach
Source: PLoS One. 2014 Mar 26;9(3):e92310. doi: 10.1371/journal.pone.0092310 (PMC3966778; doi:10.1371/journal.pone.0092310)
Supplement: Table S1 — Comparison of p-values of example pairs for different tests and both binnings in the yeast example. (DOC) [file pone.0092310.s001.doc]

| No. | Pair | ID  (Opt.) | ID  (Uni.) | Test 0  (Opt.) | Test 0 (Uni.) | Test III (Opt.) | Test III  (Uni.) |
| --- | --- | --- | --- | --- | --- | --- | --- |
| 1 | 7.9, 10.14 | 0.17 | 0.20 | <10^-7^ | <10^-7^ | <10^-7^ | <10^-7^ |
| 2 | 13.6, 10.14 | 0.073 | 0.043 | <10^-6^ | 3.9*10^-5^ | 1*10^-6^ | 4.5*10^-4^ |
| 3 | 13.6, 7.9 | -0.015 | -0.014 | <10^-6^ | <10^-6^ | 0.34 | 0.39 |
| 4 | 10.14, 16.2 | 0.023 | 0.048 | 0.009 | 4*10^-5^ | 0.01 | 2.3*10^-4^ |
| 5 | 7.8, 12.13 | 0.067 | 0.060 | <10^-6^ | 3*10^-6^ | 0.014 | 0.016 |
| 6 | 9.7, 14.11 | 0.027 | 0.063 | 0.003 | <10^-6^ | 0.005 | 8*10^-7^ |
| 7 | 9.6, 14.11 | 0.011 | 0.046 | 0.14 | 6*10^-5^ | 0.02 | 6.5*10^-4^ |
| 8 | 7.8, 14.9 | 0.046 | 0.028 | 2.8*10^-5^ | 0.0027 | 6.9*10^-5^ | 0.0053 |
| 9 | 1.2, 7.15 | 0.046 | 0.024 | 3.3*10^-5^ | 0.0068 | 8.2*10^-5^ | 0.01 |
| 10 | 10.14, 16.7 | 0.045 | 0.015 | 4.4*10^-5^ | 0.056 | 7.4*10^-5^ | 0.078 |
| 11 | 2.12, 4.23 | 0.044 | 0.04 | 4.6*10^-5^ | 1.4*10^-4^ | 8.8*10^-5^ | 3.5*10^-4^ |

Table S1. Comparison of p-values of example pairs for different tests and both binnings in the yeast example.

Opt. and Uni. refers to optimal and uniform binnings respectively.
