# Supplementary material for: Discovering Pair-Wise Genetic Interactions: An Information Theory-Based Approach
Source: PLoS One. 2014 Mar 26;9(3):e92310. doi: 10.1371/journal.pone.0092310 (PMC3966778; doi:10.1371/journal.pone.0092310)
Supplement: Table S2 — Comparison of p-values of example pairs for different tests in mouse LDL phenotype. (DOC) [file pone.0092310.s002.doc]

**Table S2. Comparison of p-values of example pairs for different tests in mouse LDL phenotype.**

| Pair | ID | Background | Test I | Test II | Test III |
| --- | --- | --- | --- | --- | --- |
| 454, 388 | 0.0398 | 8.4*10^-5^ | 1.4*10^-4^ | 1.8*10^-4^ | 1.2*10^-4^ |
| 522, 184 | 0.0404 | 7.2*10^-5^ | 7.6*10^-5^ | 6.3*10^-5^ | 1.6*10^-4^ |
| 579, 340 | 0.0419 | 4.3*10^-5^ | 4*10^-5^ | 5.1*10^-5^ | 4*10^-5^ |
| 646, 591 | 0.0402 | 7.3*10^-5^ | 1.2*10^-4^ | 1.1*10^-4^ | 1.5*10^-4^ |
| 691, 269 | 0.047 | 1.5*10^-5^ | 2.9*10^-5^ | 2.2*10^-5^ | 1.1*10^-5^ |
| 723, 9 | 0.0426 | 3.7*10^-5^ | 6.3*10^-5^ | 6.5*10^-5^ | 9*10^-5^ |
| *891*, 542 | 0.044 | 2.7*10^-5^ | 7.8*10^-5^ | 1.4*10^-4^ | 1.5*10^-4^ |
| (M)934, 96 | 0.0451 | 2.4*10^-5^ | 5.6*10^-5^ | 6.2*10^-5^ | 4*10^-5^ |
| 959, 503 | 0.0402 | 7.4*10^-5^ | 6.3*10^-5^ | 6.6*10^-5^ | 4*10^-5^ |
| 966, *878* | 0.0412 | 5.5*10^-5^ | 6.4*10^-5^ | 9*10^-5^ | 7*10^-5^ |
| 1103, 645 | 0.0448 | 2.5*10^-5^ | 3.6*10^-5^ | 4.4*10^-5^ | 4*10^-5^ |

Marker 934 has an effect on the male weight. Markers in italic (891 and 878) are the ones with the strongest effect on the LDL phenotype. Underlined markers (542 and 646) have smaller effects (p-value below 0.001).
