# Supplementary material for: Discovering Pair-Wise Genetic Interactions: An Information Theory-Based Approach
Source: PLoS One. 2014 Mar 26;9(3):e92310. doi: 10.1371/journal.pone.0092310 (PMC3966778; doi:10.1371/journal.pone.0092310)
Supplement: Table S3 — Comparison of p-values of example pairs for different tests in female weight phenotype. (DOC) [file pone.0092310.s003.doc]

**Table S3. Comparison of p-values of example pairs for different tests in female weight phenotype.**

| Pair | ID | Background | Test I | Test II | Test III |
| --- | --- | --- | --- | --- | --- |
| 502, 320 | 0.081 | 9.9*10^-5^ | 1.7*10^-4^ | 1.8*10^-4^ | 1.8*10^-4^ |
| 688, 682 | 0.082 | 9.2*10^-5^ | 1*10^-4^ | 1*10^-4^ | 7.6*10^-5^ |
| 773, **68** | 0.082 | 9.2*10^-5^ | 5*10^-4^ | 5.8*10^-4^ | 5.1*10^-4^ |
| 820, 489 | 0.082 | 9.3*10^-5^ | 1.4*10^-4^ | 1.7*10^-4^ | 1.6*10^-4^ |
| 893, 91 | 0.085 | 5.6*10^-5^ | 9.1*10^-5^ | 1.2*10^-4^ | 1*10^-4^ |
| 894, 95 | 0.091 | 2.1*10^-5^ | 5.5*10^-5^ | 8.3*10^-5^ | 2.4*10^-5^ |
| 908, 572 | 0.093 | 1.4*10^-5^ | 6.4*10^-5^ | 5.8*10^-5^ | 5.2*10^-5^ |
| 1003, 504 | 0.089 | 5.2*10^-5^ | 1.4*10^-4^ | 1.9*10^-4^ | 1.5*10^-4^ |
| 1258, 530 | 0.091 | 2.1*10^-5^ | 6.1*10^-5^ | 5.1*10^-5^ | 4.4*10^-5^ |
| 1281, 530 | 0.091 | 2.1*10^-5^ | 5.1*10^-5^ | 6*10^-5^ | 4.8*10^-5^ |

Marker 68 has an effect on the female weight.
