# Supplementary material for: Discovering Pair-Wise Genetic Interactions: An Information Theory-Based Approach
Source: PLoS One. 2014 Mar 26;9(3):e92310. doi: 10.1371/journal.pone.0092310 (PMC3966778; doi:10.1371/journal.pone.0092310)
Supplement: Table S4 — Comparison of p-values of example pairs for different tests in male weight phenotype. (DOC) [file pone.0092310.s004.doc]

**Table S4. Comparison of p-values of example pairs for different tests in male weight phenotype.**

| Pair | ID | Background | Test I | Test II | Test III |
| --- | --- | --- | --- | --- | --- |
| 135, 57(L) | 0.083 | 9.7*10^-5^ | 1.2*10^-4^ | 1.2*10^-4^ | 1*10^-4^ |
| 148, 57(L) | 0.097 | 1.2*10^-5^ | 1.7*10^-5^ | 1.3*10^-5^ | 1.2*10^-5^ |
| 388, 281 | 0.083 | 7.2*10^-5^ | 2.1*10^-4^ | 2.1*10^-4^ | 2.1*10^-4^ |
| 458, 336 | 0.085 | 6.1*10^-5^ | 2.5*10^-4^ | 2.5*10^-4^ | 2.1*10^-4^ |
| 568, 206 | 0.09 | 2.8*10^-5^ | 9.8*10^-5^ | 9.8*10^-5^ | 9.6*10^-5^ |
| 638, 388 | 0.081 | 7.8*10^-5^ | 1.4*10^-4^ | 1.6*10^-4^ | 1.4*10^-4^ |
| 697, 229 | 0.086 | 5.4*10^-5^ | 5.1*10^-5^ | 5.9*10^-5^ | 1*10^-4^ |
| 717, 681 | 0.09 | 2.9*10^-5^ | 1.1*10^-4^ | 1.1*10^-4^ | 1.1*10^-4^ |
| 746, **362** | 0.081 | 9.5*10^-5^ | 2.6*10^-4^ | 4.5*10^-4^ | 2.4*10^-4^ |
| 791, 269 | 0.084 | 6.3*10^-5^ | 9.1*10^-5^ | 1.1*10^-4^ | 4.3*10^-4^ |
| 876(L), 566 | 0.088 | 4.8*10^-5^ | 1.6*10^-4^ | 1.9*10^-4^ | 1.4*10^-4^ |
| 890(L), 367 | 0.087 | 5.1*10^-5^ | 5.8*10^-5^ | 7.9*10^-5^ | 5.6*10^-5^ |
| 920, 634 | 0.086 | 5.2*10^-5^ | 2.4*10^-4^ | 2.2*10^-4^ | 1.9*10^-4^ |
| 1021, 84(F) | 0.081 | 9.2*10^-5^ | 2.2*10^-4^ | 2.1*10^-4^ | 3*10^-4^ |
| 1192, 219 | 0.092 | 2.1*10^-5^ | 3.8*10^-5^ | 4*10^-5^ | 3.6*10^-5^ |
| 1249, 907 | 0.093 | 2*10^-5^ | 8.3*10^-5^ | 8.6*10^-5^ | 8*10^-5^ |

Indicators (F), (L), next to some markers mean that the marker has an effect on the female weight and LDL phenotype respectively. Marker 362 has an effect on the male weight.
