# Supplementary material for: Discovering Pair-Wise Genetic Interactions: An Information Theory-Based Approach
Source: PLoS One. 2014 Mar 26;9(3):e92310. doi: 10.1371/journal.pone.0092310 (PMC3966778; doi:10.1371/journal.pone.0092310)
Supplement: Table S5 — Coordinates of selected markers from the mouse example. (DOC) [file pone.0092310.s005.doc]

**Table S5. Coordinates of selected markers from the mouse example.**

| Marker | Coordinate | Marker | Coordinate |
| --- | --- | --- | --- |
| 57 | Chr. 1 - 125783518 | 68 | Chr. 1 - 149666734 |
| 84 | Chr. 1 - 183075609 | 96 | Chr. 1 - 193688025 |
| 135 | Chr. 2 - 99014733 | 148 | Chr. 2 - 114417453 |
| 269 | Chr. 3 - 130906363 | 281 | Chr. 3 - 155597929 |
| 362 | Chr. 4 - 153197886 | 367 | Chr. 5 - 13777245 |
| 388 | Chr. 5 - 61421903 | 454 | Chr. 6 - 16143378 |
| 542 | Chr. 7 - 78135647 | 566 | Chr. 7 - 109413552 |
| 591 | Chr. 8 - 32379651 | 646 | Chr. 9 - 13325606 |
| 691 | Chr. 9 - 103669734 | 742 | Chr. 10 - 124493586 |
| 773 | Chr. 11 - 42124661 | 791 | Chr. 11 - 82602424 |
| 876 | Chr. 12 - 87742858 | 878 | Chr. 12 - 87861231 |
| 890 | Chr. 12 - 113483474 | 891 | Chr. 12 - 115541493 |
| 934 | Chr. 13 - 85537336 | 966 | Chr. 14 - 42571526 |
| 1021 | Chr. 15 - 32311930 |  |  |
