# Supplementary material for: Discovering Pair-Wise Genetic Interactions: An Information Theory-Based Approach
Source: PLoS One. 2014 Mar 26;9(3):e92310. doi: 10.1371/journal.pone.0092310 (PMC3966778; doi:10.1371/journal.pone.0092310)
Supplement: Code S1 — This file contains Matlab scripts that will enable readers to reproduce results presented in the paper and apply presented tools to their data. (ZIP) [file pone.0092310.s006.zip › Matlab Code/readme.pdf]

File "Simu_1" contains a Matlab script generating figures similar to Figure 3 and 4 presented in the paper. In order to use it, one has to set up several parameters (MAFs, penetrance function, number of cases and controls). They are defined and explained at the beginning of the file.

The script uses several other Matlab functions which are described in the corresponding files. User can utilize them to compute information-theoretic measures (entropy, conditional entropy, mutual information, conditional mutual information, information distance and interaction information) of his/her data (see lines 60 – 70 of “Simu_1” for an example).

Additionally, we attach function “Test3” which performs permutation Test III described in the paper.
